# (5-*n*-Butyl-10,20-diiso­butyl­porphyrin­ato)nickel(II)

**DOI:** 10.1107/S1600536814012884

**Published:** 2014-06-07

**Authors:** Mathias O. Senge, Katja Dahms

**Affiliations:** a SFI Tetrapyrrole Laboratory, School of Chemistry, Trinity College Dublin, Dublin 2, Ireland

## Abstract

The asymmetric unit of the title compound, [Ni(C_32_H_36_N_4_)], contains two independent mol­ecules exhibiting an overall ruffled conformation of the porphyrin macrocycle and differing mainly in the positions of the methyl groups. The average Ni—N bond lengths are 1.912 (2) and 1.910 (2) Å in the two mol­ecules. The mol­ecules form a closely spaced lattice structure in which neighbouring porphyrins are oriented in a nearly perpendicular fashion to each other. The compound was prepared *via* nucleophilic substitution of (5,15-diiso­butyl­porphyrinato)nickel(II) with *n*-butyl­lithium.

## Related literature   

For the conformations of porphyrins, see: Scheidt & Lee (1987 ▶); Jentzen *et al.* (1997 ▶); Senge (2006 ▶). For the synthesis of related compounds, see: Senge (2005 ▶); Wiehe *et al.* (2005 ▶). For the handling of crystals, see: Hope (1994 ▶). For related structures, see: Senge *et al.* (1999 ▶); Senge (2012 ▶).
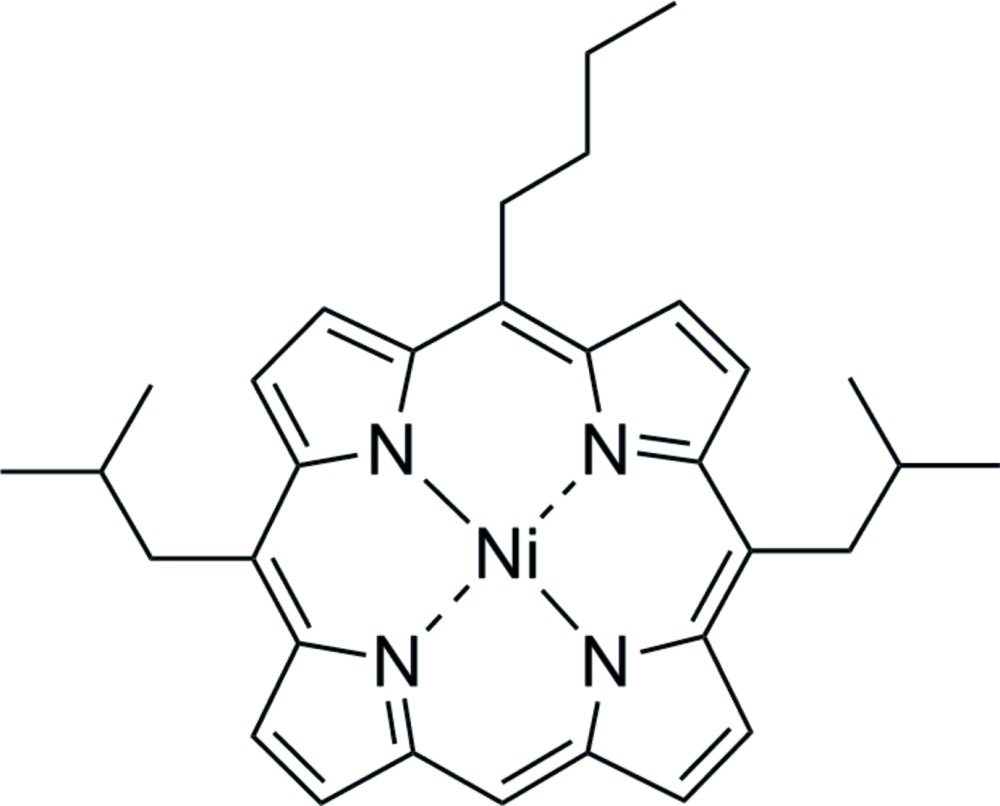


## Experimental   

### 

#### Crystal data  

[Ni(C_32_H_36_N_4_)]
*M* *_r_* = 535.34
Triclinic, 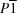

*a* = 10.2557 (14) Å
*b* = 11.3234 (15) Å
*c* = 23.220 (3) Å
α = 87.474 (2)°
β = 81.501 (2)°
γ = 89.955 (2)°
*V* = 2664.2 (6) Å^3^
*Z* = 4
Mo *K*α radiation
μ = 0.76 mm^−1^
*T* = 90 K
0.40 × 0.30 × 0.28 mm

#### Data collection  

Bruker SMART CCD area-detector diffractometer
Absorption correction: multi-scan (*SADABS*; Bruker, 2005 ▶) *T*_min_ = 0.752, *T*_max_ = 0.816
34414 measured reflections
12344 independent reflections
9370 reflections with *I* > 2σ(*I*)
*R*_int_ = 0.035

#### Refinement  

*R*[*F* ^2^ > 2σ(*F* ^2^)] = 0.037
*wR*(*F* ^2^) = 0.106
*S* = 1.04
12344 reflections
677 parameters
H-atom parameters constrained
Δρ_max_ = 0.58 e Å^−3^
Δρ_min_ = −0.47 e Å^−3^

### 

Data collection: *SMART* (Bruker, 2005 ▶); cell refinement: *SAINT* (Bruker, 2005 ▶); data reduction: *SAINT*; program(s) used to solve structure: *SIR92* (Altomare *et al.*, 1994 ▶); program(s) used to refine structure: *SHELXL97* (Sheldrick, 2008 ▶); molecular graphics: *XP* in *SHELXTL* (Sheldrick, 2008 ▶) and *Mercury* (Macrae *et al.*, 2006 ▶); software used to prepare material for publication: *SHELXTL-Plus* (Sheldrick, 2008 ▶).

## Supplementary Material

Crystal structure: contains datablock(s) I, New_Global_Publ_Block. DOI: 10.1107/S1600536814012884/cv5460sup1.cif

Structure factors: contains datablock(s) I. DOI: 10.1107/S1600536814012884/cv5460Isup2.hkl

CCDC reference: 1006411

Additional supporting information:  crystallographic information; 3D view; checkCIF report

## Figures and Tables

**Fig. 1.**
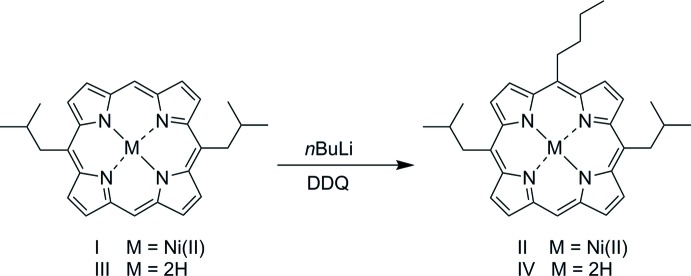
Synthesis of the title compound.

**Fig. 2.**
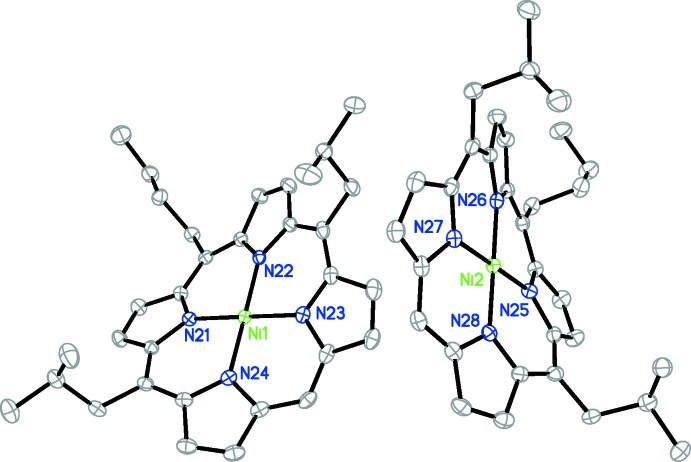
A content of the asymmetric unit of (II), showing the atomic labels for Ni and N atoms. Displacement ellipsoids are drawn at the 50% probabilty level. H atoms have been omitted for clarity.

**Fig. 3.**
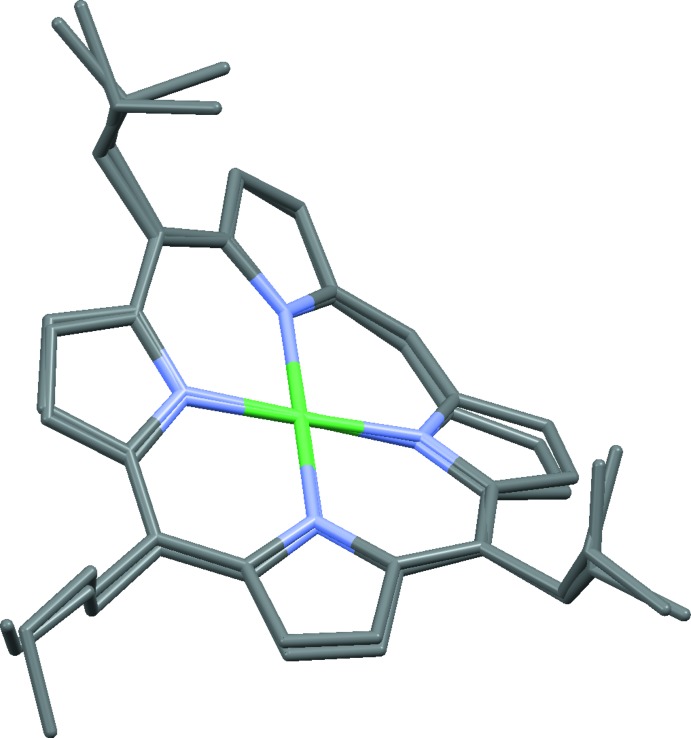
An overlay of the two independent molecules showing the differences in their conformations. Prepared with Mercury (Macrae et al., 2006).

**Fig. 4.**
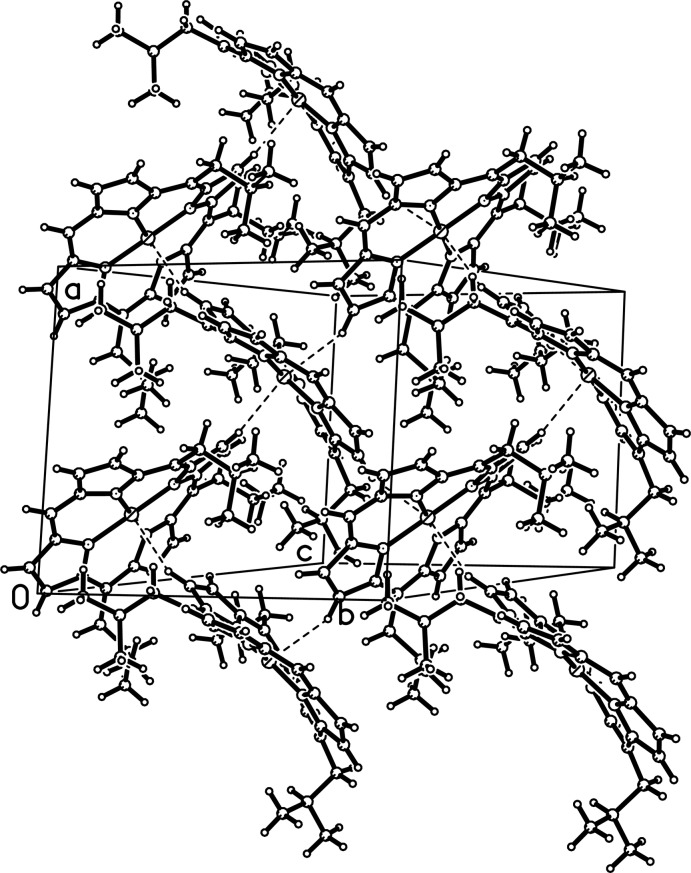
View of the molecular arrangement of (II) in the crystal. Dashed lines indicate short Ni···H separations.

## supplementary crystallographic information

### S1. Comment

Reaction of (5,15-diisobutylporphyrinato)nickel(II), (I), with *n*-butyllithium yielded the title compound, (II) (Fig. 1). The synthesis followed the general strategy outlined earlier (Senge, 2005). Similarly, the respective free base (IV) could be prepared *via* reaction of (III) with *n*-butyllithium. Crystals suitable for single-crystal X-ray crystallography were grown by liquid diffusion of methanol into a solution of (II) in methylene chloride.

The compound crystallized with two crystallographically independent molecules in the unit cell. A top view of the molecular structure is shown in Fig. 2. All three alkyl side chains point towards the same face of the molecules. The compound is characterized by an overall ruffled conformation (Fig. 3), as indicated by mean displacements of the C_m_ atoms by about 0.6 Å (Table 1).

The molecules form a closely spaced lattice structure in which neighbouring porphyrin are oriented in a perpendicular fashion to each other. The edge of one molecule points with a β-H atoms towards the Ni^II^ centre of the next molecule. The H···Ni separations are small, with H22A···Ni1 = 2.593 Å, H12A···Ni2^i^ = 2.667 Å, H32A···Ni1^ii^ = 2.757 Å and H2A···Ni2^iii^ = 2.635 Å [symmetry codes: (i) *x*, *y*+1, *z*; (ii) *x*-1, *y*-1, *z*; (iii) *x*+1, *y*, *z*]. Thus, the two faces of each macrocycle core are blocked by a neighbouring macrocycle in edge-on fashion (Fig. 4).

A conformational analys was performed using the NSD (normal structural decomposition method) developed by Shelnutt and co-workers (Jentzen *et al.*, 1997). Both molecules exhibit the same conformation with only minor differences in conformational parameters and bond lengths and angles. The conformation is characterized by a significant degree of *ruf* distortion which is in line with expectations for a sterically unhindered Ni(II) *meso*-alkylporphyrin. The degree of displacement of the C_m_ atoms, which is characteristic for a ruffled conformation, is similar to that found for symmetric (5,10,15,20-tetraalkylporphyrinato)nickel(II) systems (Senge *et al.*, 1999). Only negligible differences are found in displacements for substituted *versus* unsubstituted *meso* carbon atoms. One of the molecules exhibits a small degree of *sad* distortion and both show a contribution from *bre* in-plane distortion. Note, that compound (I), *i.e.* the *meso* disubstituted precursor compound, exhibits a planar macrocycle (Senge, 2012).

### S2. Experimental

The title compound, (II), was prepared *via* reaction of compound (I) with *n*-butyllithium. A similar reaction of the free base porphyrin (III) yielded 5-*n*-butyl-10,15-diisobutylporphyrin, (IV).

Synthesis of (5-*n*-butyl-10,20-diisobutylporphyrinato)nickel(II), (II):

(5,15-Diisobutylporphyrinato)nickel(II), (I) (Wiehe *et al.*, 2005), (1 eq.) was placed in a Schlenk flask and dissolved in dry THF under argon. The solution was cooled down to -78 °C. Then *n*-butyllithium (20 eq., 2.5 *M* solution in *n*-hexane) and *N,N,N',N'*-tetramethylethylendiamine (3.3 eq.) were added dropwise. The mixture was allowed to warm up to room temperature and was stirred for 30 additional minutes. The reaction was quenched with 3 ml water and stirred for another 15 min. DDQ (5.5 eq.) was added. Stirring was continued for 15 min followed by filtration of the reaction mixture through 200 ml silica gel and washing with dichloromethane. The eluted porphyrin fractions were evaporated to dryness. The residue was dissolved in dichloromethane and purified *via* column chromatography on silica gel with dichloromethane: *n*-hexane (1:3, *v*/*v*, h = 48 cm, ø = 3 cm). The product was obtained as purple crystals after recrystallization from dichloromethane/methanol 27.2 mg (0.05 mmol, 28%): *Mp* > 310 °C, *R*_f_=0.7 (CH_2_Cl_2_: *n*-hexane, 1:3, *v*/*v*); ^1^H NMR (300 MHz, CDCl_3_): *δ* = 0.88 (d, 12H, ^3^*J* = 6.6 Hz, CH(C*H*_3_)_2_), 1.05 (t, 3H, ^3^*J* = 7.3 Hz, CH_2_C*H*_3_), 1.60 (m, 2H, C*H*_2_CH_3_), 2.30 (m, 4H, C*H*(CH_3_)_2_ + CH_2_C*H*_2_CH_2_), 4.56 (m, 6H, C*H*_2_CH_2_CH_2_ + C*H*_2_CH), 9.05 (d, 2H, ^3^*J* = 4.9 Hz, *H*_β_), 9.34 (d, 2H, *^3^J* = 4.9 Hz, *H*_β_), 9.36 (s, 4H, *H*_β_), 9.49 p.p.m. (s, 1H, *H_meso_*); ^13^C NMR (63 MHz, CDCl_3_): *δ* = 14.0, 23.0, 23.5, 34.0, 34.5, 39.6, 42.3, 102.6, 115.8, 117.9, 129.6, 130.2, 130.5, 132.0, 140.7, 141.6, 142.2, 142.3 p.p.m.; UV/vis (CH_2_Cl_2_): *λ*_max_ (lg *ε*) = 411 (5.55), 528 nm (4.29); MS (EI, 70 eV): *m/z* (%) = 534 (89) [*M*^+^], 491 (100) [*M*^+^—C_3_H_7_], 448 (18) [*M*^+^—C_6_H_14_], 267 (2) [*M*^2+^]; HRMS (ES+): [C_32_H_36_N_4_Ni]: calcd. 534.2293, found 534.2300.

Synthesis of 5-*n*-butyl-10,20-diisobutylporphyrin, (IV):

5,15-Diisobutylporphyrin, (III) (Wiehe *et al.*, 2005) (1 eq.), was placed in a Schlenk flask and dissolved in dry THF under argon. The solution was cooled down to -78 °C. Then *n*-butyllithium (20 eq., 2.5 *M* solution in *n*-hexane) and *N,N,N',N'*-tetramethylethylendiamine (3.3 eq.) were added dropwise. The mixture was allowed to warm up to room temperature and was stirred for 30 additional minutes. The reaction was quenched with 3 ml water and stirred for another 15 min. DDQ (5.5 eq.) was added. Stirring was continued for 15 min followed by filtration of the reaction mixture through 200 ml silica gel and washing with dichloromethane. The eluted porphyrin fractions were evaporated to dryness. The residue was dissolved in dichloromethane and purified *via* column chromatography on silica gel with dichloromethane: *n*-hexane (1:1, *v*/*v*, h = 45 cm, ø = 3 cm). The product was obtained as purple crystals after recrystallization from dichloromethane/methanol (14.2 mg, 0.03 mmol, 15%): Mp 195 °C, *R*_f_ = 0.5 (CH_2_Cl_2_: *n*-hexane, 1:1, *v*/*v*); ^1^H NMR (300 MHz, CDCl_3_): *δ* = -2.83 (s, 2H, N*H*), 0.97 (t, 3H, ^3^J = 7.3 Hz, CH_2_C*H*_3_), 1.21 (d, 12H, ^3^J = 6.6 Hz, CH(C*H*_3_)_2_), 1. 87 (m, 2H, C*H*_2_CH_3_), 2.58 (m, 2H, CH_2_C*H*_2_CH_2_), 2.79 (m, 2H, C*H*(CH_3_)_2_), 4.84 (d, 4H, ^3^J = 7.3 Hz, C*H*_2_CH), 5.08 (t, 2H, ^3^J = 8.0 Hz, C*H*_2_CH_2_CH_2_), 9.29 (d, 2H, ^3^J = 4.7 Hz, 13,17-*H*_β_), 9.51 (dd, 4H, ^3^J = 4.9 Hz, ^3^J = 4.7 Hz, 2,8,12,18-*H*_β_), 9.61 (d, 2H, ^3^J = 4.9 Hz, 3,7-*H*_β_), 9.96 p.p.m. (s, 1H, *H_meso_*); ^13^C NMR (75 MHz, CDCl_3_): *δ* = 14.2, 23.4, 23.8, 36.0, 36.7, 41.2, 43.4, 53.4, 103.1, 117.4, 119.9, 128.5, 128.7, 131.1 p.p.m.; UV/vis (CH_2_Cl_2_): *λ*_max_ (lg *ε*) = 413 (4.22), 512 (3.95), 542 (3.63), 588 (3.32), 645 nm (3.16); MS (EI, 70 eV): *m/z* (%)= 478 (52) [*M*^+^], 435 (100) [*M*^+^—C_3_H_7_], 392 (12) [*M*^+^—C_6_H_14_], 239 (3) [*M*^2+^]; HRMS (ES+): [C_32_H_38_N_4_+H]: calcd. 479.3175, found 479.3142.

### Crystal data

**Table:**

[Ni(C_32_H_36_N_4_)]	*Z* = 4
*M**_r_* = 535.34	*F*(000) = 1136
Triclinic, *P*1	*D*_x_ = 1.335 Mg m^−^^3^
Hall symbol: -P 1	Mo *K*α radiation, λ = 0.71073 Å
*a* = 10.2557 (14) Å	Cell parameters from 5659 reflections
*b* = 11.3234 (15) Å	θ = 5.3–55.1°
*c* = 23.220 (3) Å	µ = 0.76 mm^−^^1^
α = 87.474 (2)°	*T* = 90 K
β = 81.501 (2)°	Block, red
γ = 89.955 (2)°	0.40 × 0.30 × 0.28 mm
*V* = 2664.2 (6) Å^3^	

### Data collection

**Table:**

Bruker SMART CCD area-detector diffractometer	12344 independent reflections
Radiation source: sealed tube	9370 reflections with *I* > 2σ(*I*)
Graphite monochromator	*R*_int_ = 0.035
phi and ω scans	θ_max_ = 27.7°, θ_min_ = 0.9°
Absorption correction: multi-scan (*SADABS*; Bruker, 2005)	*h* = −13→13
*T*_min_ = 0.752, *T*_max_ = 0.816	*k* = −14→14
34414 measured reflections	*l* = −30→30

### Refinement

**Table:**

Refinement on *F*^2^	Primary atom site location: structure-invariant direct methods
Least-squares matrix: full	Secondary atom site location: difference Fourier map
*R*[*F*^2^ > 2σ(*F*^2^)] = 0.037	Hydrogen site location: inferred from neighbouring sites
*wR*(*F*^2^) = 0.106	H-atom parameters constrained
*S* = 1.04	*w* = 1/[σ^2^(*F*_o_^2^) + (0.0531*P*)^2^ + 1.1259*P*] where *P* = (*F*_o_^2^ + 2*F*_c_^2^)/3
12344 reflections	(Δ/σ)_max_ = 0.001
677 parameters	Δρ_max_ = 0.58 e Å^−^^3^
0 restraints	Δρ_min_ = −0.47 e Å^−^^3^

### Special details

**Table:**

Geometry. All e.s.d.'s (except the e.s.d. in the dihedral angle between two l.s. planes) are estimated using the full covariance matrix. The cell e.s.d.'s are taken into account individually in the estimation of e.s.d.'s in distances, angles and torsion angles; correlations between e.s.d.'s in cell parameters are only used when they are defined by crystal symmetry. An approximate (isotropic) treatment of cell e.s.d.'s is used for estimating e.s.d.'s involving l.s. planes.
Refinement. Refinement of *F*^2^ against ALL reflections. The weighted *R*-factor *wR* and goodness of fit *S* are based on *F*^2^, conventional *R*-factors *R* are based on *F*, with *F* set to zero for negative *F*^2^. The threshold expression of *F*^2^ > σ(*F*^2^) is used only for calculating *R*-factors(gt) *etc*. and is not relevant to the choice of reflections for refinement. *R*-factors based on *F*^2^ are statistically about twice as large as those based on *F*, and *R*- factors based on ALL data will be even larger. The out carbon atom of the butyl chains shows some degree of thermal librational movement.

### Fractional atomic coordinates and isotropic or equivalent isotropic displacement parameters (Å^2^)

**Table:**

	*x*	*y*	*z*	*U*_iso_*/*U*_eq_	
Ni1	0.31532 (2)	0.93466 (2)	0.278602 (11)	0.01394 (7)	
N21	0.20303 (16)	1.06325 (15)	0.26337 (7)	0.0155 (3)	
N22	0.38963 (16)	0.93563 (14)	0.19812 (7)	0.0149 (3)	
N23	0.42627 (17)	0.80379 (15)	0.29343 (7)	0.0171 (4)	
N24	0.24418 (16)	0.93538 (15)	0.35971 (7)	0.0167 (4)	
C1	0.1363 (2)	1.13695 (18)	0.30319 (9)	0.0169 (4)	
C2	0.0596 (2)	1.2206 (2)	0.27413 (10)	0.0222 (5)	
H2A	0.0071	1.2822	0.2917	0.027*	
C3	0.0760 (2)	1.19549 (19)	0.21742 (9)	0.0213 (5)	
H3A	0.0340	1.2335	0.1879	0.026*	
C4	0.1687 (2)	1.10054 (18)	0.20991 (9)	0.0173 (4)	
C5	0.2273 (2)	1.06158 (18)	0.15644 (9)	0.0172 (4)	
C6	0.3379 (2)	0.98923 (18)	0.15189 (9)	0.0171 (4)	
C7	0.4241 (2)	0.97116 (19)	0.09862 (9)	0.0213 (4)	
H7A	0.4095	0.9963	0.0605	0.026*	
C8	0.5299 (2)	0.9117 (2)	0.11266 (10)	0.0230 (5)	
H8A	0.6055	0.8904	0.0864	0.028*	
C9	0.5077 (2)	0.88651 (18)	0.17414 (9)	0.0184 (4)	
C10	0.5865 (2)	0.81180 (18)	0.20397 (10)	0.0202 (4)	
C11	0.5393 (2)	0.76651 (18)	0.25950 (10)	0.0196 (4)	
C12	0.5926 (2)	0.6651 (2)	0.28770 (10)	0.0252 (5)	
H12A	0.6718	0.6249	0.2741	0.030*	
C13	0.5091 (2)	0.6381 (2)	0.33694 (10)	0.0252 (5)	
H13A	0.5155	0.5728	0.3635	0.030*	
C14	0.4087 (2)	0.72641 (18)	0.34176 (10)	0.0203 (4)	
C15	0.3205 (2)	0.74236 (19)	0.39121 (9)	0.0217 (5)	
H15A	0.3065	0.6795	0.4200	0.026*	
C16	0.2515 (2)	0.84554 (19)	0.40095 (9)	0.0200 (4)	
C17	0.1902 (2)	0.8800 (2)	0.45693 (10)	0.0252 (5)	
H17A	0.1794	0.8323	0.4920	0.030*	
C18	0.1508 (2)	0.9926 (2)	0.45073 (9)	0.0237 (5)	
H18A	0.1114	1.0406	0.4810	0.028*	
C19	0.1792 (2)	1.02699 (19)	0.38957 (9)	0.0188 (4)	
C20	0.1331 (2)	1.12786 (19)	0.36344 (9)	0.0183 (4)	
C5A	0.1812 (2)	1.11047 (19)	0.10108 (9)	0.0201 (4)	
H5AA	0.0859	1.1279	0.1095	0.024*	
H5AB	0.1930	1.0492	0.0716	0.024*	
C5B	0.2548 (2)	1.2228 (2)	0.07539 (9)	0.0245 (5)	
H5BA	0.2374	1.2861	0.1036	0.029*	
H5BB	0.3507	1.2072	0.0696	0.029*	
C5C	0.2146 (3)	1.2659 (2)	0.01739 (10)	0.0291 (5)	
H5CA	0.2258	1.2008	−0.0099	0.035*	
H5CB	0.1202	1.2874	0.0237	0.035*	
C5D	0.2952 (3)	1.3722 (2)	−0.00990 (12)	0.0445 (7)	
H5DA	0.2657	1.3966	−0.0469	0.067*	
H5DB	0.3887	1.3509	−0.0171	0.067*	
H5DC	0.2832	1.4375	0.0167	0.067*	
C10A	0.7199 (2)	0.7755 (2)	0.17288 (10)	0.0238 (5)	
H10A	0.7107	0.7548	0.1327	0.029*	
H10B	0.7495	0.7039	0.1932	0.029*	
C10B	0.8251 (2)	0.8721 (2)	0.17017 (10)	0.0243 (5)	
H10C	0.7920	0.9450	0.1513	0.029*	
C10C	0.8518 (3)	0.9024 (3)	0.23078 (12)	0.0387 (6)	
H10D	0.7710	0.9328	0.2531	0.058*	
H10E	0.9213	0.9627	0.2272	0.058*	
H10F	0.8801	0.8312	0.2510	0.058*	
C10D	0.9512 (2)	0.8344 (2)	0.13253 (11)	0.0321 (6)	
H10G	1.0186	0.8962	0.1314	0.048*	
H10H	0.9334	0.8223	0.0929	0.048*	
H10I	0.9829	0.7605	0.1490	0.048*	
C20A	0.0701 (2)	1.2261 (2)	0.40013 (9)	0.0223 (5)	
H20A	0.0573	1.1980	0.4416	0.027*	
H20B	−0.0181	1.2424	0.3892	0.027*	
C20B	0.1496 (2)	1.3423 (2)	0.39398 (10)	0.0252 (5)	
H20C	0.1469	1.3783	0.3541	0.030*	
C20C	0.2933 (2)	1.3236 (2)	0.40132 (13)	0.0377 (6)	
H20D	0.3380	1.4004	0.3996	0.057*	
H20E	0.3364	1.2756	0.3700	0.057*	
H20F	0.2980	1.2829	0.4391	0.057*	
C20D	0.0830 (3)	1.4269 (2)	0.43839 (11)	0.0325 (6)	
H20G	−0.0098	1.4361	0.4335	0.049*	
H20H	0.1274	1.5040	0.4323	0.049*	
H20I	0.0886	1.3950	0.4779	0.049*	
Ni2	0.80710 (2)	0.42660 (2)	0.286765 (11)	0.01391 (7)	
N25	0.69867 (16)	0.30199 (15)	0.26770 (7)	0.0161 (3)	
N26	0.88415 (16)	0.44790 (14)	0.20710 (7)	0.0156 (3)	
N27	0.91480 (17)	0.55279 (15)	0.30578 (7)	0.0170 (4)	
N28	0.72924 (16)	0.40601 (15)	0.36676 (7)	0.0159 (3)	
C21	0.6345 (2)	0.21597 (18)	0.30528 (9)	0.0178 (4)	
C22	0.5631 (2)	0.1380 (2)	0.27340 (10)	0.0230 (5)	
H22A	0.5135	0.0701	0.2890	0.028*	
C23	0.5797 (2)	0.1792 (2)	0.21761 (10)	0.0233 (5)	
H23A	0.5404	0.1483	0.1869	0.028*	
C24	0.6680 (2)	0.27838 (18)	0.21314 (9)	0.0178 (4)	
C25	0.7264 (2)	0.33368 (18)	0.16104 (9)	0.0179 (4)	
C26	0.8343 (2)	0.40864 (18)	0.15906 (9)	0.0171 (4)	
C27	0.9212 (2)	0.44412 (19)	0.10688 (9)	0.0215 (4)	
H27A	0.9075	0.4318	0.0680	0.026*	
C28	1.0262 (2)	0.4983 (2)	0.12346 (9)	0.0231 (5)	
H28A	1.1021	0.5274	0.0984	0.028*	
C29	1.0023 (2)	0.50376 (18)	0.18557 (9)	0.0179 (4)	
C30	1.0804 (2)	0.56742 (18)	0.21814 (9)	0.0191 (4)	
C31	1.0312 (2)	0.59727 (18)	0.27420 (9)	0.0185 (4)	
C32	1.0819 (2)	0.68996 (19)	0.30550 (10)	0.0231 (5)	
H32A	1.1620	0.7326	0.2941	0.028*	
C33	0.9949 (2)	0.70478 (19)	0.35376 (10)	0.0232 (5)	
H33A	0.9997	0.7625	0.3818	0.028*	
C34	0.8936 (2)	0.61787 (18)	0.35511 (9)	0.0195 (4)	
C35	0.7992 (2)	0.59113 (19)	0.40261 (9)	0.0201 (4)	
H35A	0.7825	0.6470	0.4321	0.024*	
C36	0.7282 (2)	0.48733 (19)	0.40918 (9)	0.0191 (4)	
C37	0.6564 (2)	0.4413 (2)	0.46314 (9)	0.0229 (5)	
H37A	0.6380	0.4819	0.4983	0.028*	
C38	0.6200 (2)	0.3299 (2)	0.45469 (9)	0.0230 (5)	
H38A	0.5748	0.2760	0.4833	0.028*	
C39	0.6624 (2)	0.30757 (19)	0.39415 (9)	0.0182 (4)	
C40	0.6267 (2)	0.20995 (19)	0.36567 (9)	0.0191 (4)	
C25A	0.6851 (2)	0.29766 (19)	0.10455 (9)	0.0215 (5)	
H25A	0.6994	0.3650	0.0756	0.026*	
H25B	0.5897	0.2787	0.1112	0.026*	
C25B	0.7613 (3)	0.1905 (2)	0.07968 (10)	0.0288 (5)	
H25C	0.8568	0.2089	0.0741	0.035*	
H25D	0.7451	0.1229	0.1083	0.035*	
C25C	0.7236 (3)	0.1542 (2)	0.02160 (10)	0.0322 (6)	
H25E	0.7686	0.0793	0.0107	0.039*	
H25F	0.6274	0.1391	0.0268	0.039*	
C25D	0.7587 (3)	0.2455 (2)	−0.02770 (11)	0.0361 (6)	
H25G	0.7413	0.2131	−0.0643	0.054*	
H25H	0.8523	0.2663	−0.0309	0.054*	
H25I	0.7052	0.3164	−0.0198	0.054*	
C30A	1.2157 (2)	0.60893 (19)	0.18913 (10)	0.0229 (5)	
H30A	1.2502	0.6669	0.2139	0.027*	
H30B	1.2074	0.6497	0.1513	0.027*	
C30B	1.3145 (2)	0.5075 (2)	0.17884 (10)	0.0244 (5)	
H30C	1.2753	0.4475	0.1560	0.029*	
C30C	1.4416 (2)	0.5535 (2)	0.14223 (11)	0.0319 (6)	
H30D	1.4216	0.5897	0.1055	0.048*	
H30E	1.5027	0.4877	0.1340	0.048*	
H30F	1.4821	0.6126	0.1637	0.048*	
C30D	1.3408 (3)	0.4469 (2)	0.23549 (12)	0.0364 (6)	
H30G	1.2589	0.4113	0.2560	0.055*	
H30H	1.3735	0.5051	0.2600	0.055*	
H30I	1.4070	0.3851	0.2271	0.055*	
C40A	0.5723 (2)	0.09968 (19)	0.40010 (9)	0.0216 (5)	
H40A	0.4814	0.0860	0.3922	0.026*	
H40B	0.5673	0.1135	0.4422	0.026*	
C40B	0.6534 (2)	−0.0122 (2)	0.38638 (10)	0.0238 (5)	
H40C	0.6361	−0.0393	0.3478	0.029*	
C40C	0.8014 (2)	0.0077 (2)	0.38293 (10)	0.0270 (5)	
H40D	0.8314	0.0663	0.3514	0.040*	
H40E	0.8477	−0.0670	0.3751	0.040*	
H40F	0.8203	0.0366	0.4200	0.040*	
C40D	0.6058 (2)	−0.1096 (2)	0.43290 (11)	0.0295 (5)	
H40G	0.5097	−0.1170	0.4369	0.044*	
H40H	0.6312	−0.0891	0.4703	0.044*	
H40I	0.6463	−0.1850	0.4212	0.044*	

### Atomic displacement parameters (Å^2^)

**Table:**

	*U*^11^	*U*^22^	*U*^33^	*U*^12^	*U*^13^	*U*^23^
Ni1	0.01368 (13)	0.01396 (13)	0.01492 (13)	0.00002 (10)	−0.00454 (10)	−0.00064 (10)
N21	0.0155 (8)	0.0169 (8)	0.0147 (8)	0.0000 (7)	−0.0038 (7)	−0.0014 (7)
N22	0.0138 (8)	0.0135 (8)	0.0178 (8)	−0.0006 (6)	−0.0033 (7)	−0.0019 (7)
N23	0.0169 (9)	0.0161 (8)	0.0196 (9)	−0.0001 (7)	−0.0068 (7)	−0.0005 (7)
N24	0.0157 (8)	0.0185 (9)	0.0166 (8)	−0.0013 (7)	−0.0053 (7)	0.0007 (7)
C1	0.0152 (10)	0.0182 (10)	0.0172 (10)	−0.0003 (8)	−0.0021 (8)	−0.0013 (8)
C2	0.0209 (11)	0.0239 (11)	0.0222 (11)	0.0062 (9)	−0.0042 (9)	−0.0026 (9)
C3	0.0213 (11)	0.0240 (11)	0.0199 (11)	0.0045 (9)	−0.0078 (9)	0.0009 (9)
C4	0.0158 (10)	0.0187 (10)	0.0181 (10)	−0.0003 (8)	−0.0051 (8)	−0.0013 (8)
C5	0.0177 (10)	0.0175 (10)	0.0171 (10)	−0.0035 (8)	−0.0049 (8)	−0.0001 (8)
C6	0.0197 (10)	0.0164 (10)	0.0161 (10)	−0.0031 (8)	−0.0047 (8)	−0.0027 (8)
C7	0.0233 (11)	0.0251 (11)	0.0160 (10)	0.0005 (9)	−0.0031 (8)	−0.0035 (9)
C8	0.0204 (11)	0.0244 (11)	0.0234 (11)	0.0000 (9)	0.0010 (9)	−0.0054 (9)
C9	0.0171 (10)	0.0167 (10)	0.0214 (10)	−0.0018 (8)	−0.0018 (8)	−0.0043 (8)
C10	0.0170 (10)	0.0166 (10)	0.0281 (12)	0.0002 (8)	−0.0054 (9)	−0.0057 (9)
C11	0.0159 (10)	0.0172 (10)	0.0273 (11)	0.0002 (8)	−0.0080 (8)	−0.0039 (9)
C12	0.0201 (11)	0.0225 (11)	0.0350 (13)	0.0044 (9)	−0.0107 (10)	−0.0019 (10)
C13	0.0285 (12)	0.0189 (11)	0.0305 (12)	0.0014 (9)	−0.0124 (10)	0.0023 (9)
C14	0.0214 (11)	0.0170 (10)	0.0249 (11)	−0.0013 (8)	−0.0124 (9)	0.0015 (9)
C15	0.0250 (11)	0.0203 (11)	0.0214 (11)	−0.0057 (9)	−0.0103 (9)	0.0061 (9)
C16	0.0200 (11)	0.0236 (11)	0.0174 (10)	−0.0051 (9)	−0.0072 (8)	0.0030 (8)
C17	0.0248 (12)	0.0323 (13)	0.0185 (11)	−0.0040 (10)	−0.0045 (9)	0.0042 (9)
C18	0.0218 (11)	0.0318 (13)	0.0176 (11)	−0.0024 (9)	−0.0026 (9)	−0.0009 (9)
C19	0.0167 (10)	0.0240 (11)	0.0159 (10)	−0.0047 (8)	−0.0034 (8)	−0.0018 (8)
C20	0.0154 (10)	0.0223 (11)	0.0172 (10)	−0.0013 (8)	−0.0015 (8)	−0.0026 (8)
C5A	0.0227 (11)	0.0238 (11)	0.0149 (10)	0.0013 (9)	−0.0059 (8)	−0.0014 (8)
C5B	0.0323 (13)	0.0225 (11)	0.0191 (11)	0.0000 (10)	−0.0054 (9)	0.0010 (9)
C5C	0.0358 (13)	0.0311 (13)	0.0195 (11)	0.0086 (11)	−0.0021 (10)	0.0028 (10)
C5D	0.064 (2)	0.0369 (15)	0.0287 (14)	0.0055 (14)	0.0025 (13)	0.0106 (12)
C10A	0.0184 (11)	0.0229 (11)	0.0298 (12)	0.0037 (9)	−0.0016 (9)	−0.0036 (9)
C10B	0.0195 (11)	0.0220 (11)	0.0303 (12)	0.0015 (9)	−0.0011 (9)	0.0016 (9)
C10C	0.0239 (13)	0.0538 (17)	0.0392 (15)	−0.0107 (12)	−0.0042 (11)	−0.0125 (13)
C10D	0.0231 (12)	0.0343 (14)	0.0364 (14)	−0.0018 (10)	0.0037 (10)	−0.0001 (11)
C20A	0.0204 (11)	0.0286 (12)	0.0176 (10)	0.0036 (9)	−0.0006 (8)	−0.0046 (9)
C20B	0.0267 (12)	0.0270 (12)	0.0223 (11)	0.0047 (10)	−0.0042 (9)	−0.0054 (9)
C20C	0.0276 (13)	0.0355 (14)	0.0513 (17)	0.0021 (11)	−0.0066 (12)	−0.0153 (13)
C20D	0.0346 (14)	0.0338 (14)	0.0322 (13)	0.0092 (11)	−0.0117 (11)	−0.0116 (11)
Ni2	0.01358 (13)	0.01427 (13)	0.01468 (13)	−0.00048 (10)	−0.00432 (10)	−0.00196 (10)
N25	0.0145 (8)	0.0184 (9)	0.0159 (8)	−0.0016 (7)	−0.0032 (7)	−0.0024 (7)
N26	0.0162 (8)	0.0136 (8)	0.0180 (8)	0.0005 (7)	−0.0052 (7)	−0.0010 (7)
N27	0.0171 (9)	0.0164 (8)	0.0186 (9)	0.0012 (7)	−0.0059 (7)	−0.0022 (7)
N28	0.0145 (8)	0.0177 (8)	0.0161 (8)	0.0027 (7)	−0.0043 (7)	−0.0015 (7)
C21	0.0160 (10)	0.0164 (10)	0.0213 (10)	−0.0012 (8)	−0.0031 (8)	−0.0011 (8)
C22	0.0231 (11)	0.0234 (11)	0.0235 (11)	−0.0084 (9)	−0.0070 (9)	0.0002 (9)
C23	0.0238 (11)	0.0244 (11)	0.0235 (11)	−0.0054 (9)	−0.0088 (9)	−0.0027 (9)
C24	0.0170 (10)	0.0193 (10)	0.0183 (10)	0.0003 (8)	−0.0060 (8)	−0.0022 (8)
C25	0.0197 (10)	0.0174 (10)	0.0177 (10)	0.0034 (8)	−0.0059 (8)	−0.0015 (8)
C26	0.0184 (10)	0.0169 (10)	0.0166 (10)	0.0021 (8)	−0.0051 (8)	0.0007 (8)
C27	0.0235 (11)	0.0246 (11)	0.0164 (10)	0.0005 (9)	−0.0031 (8)	−0.0005 (9)
C28	0.0236 (11)	0.0250 (11)	0.0198 (11)	−0.0003 (9)	−0.0007 (9)	0.0006 (9)
C29	0.0179 (10)	0.0154 (10)	0.0202 (10)	0.0010 (8)	−0.0028 (8)	−0.0012 (8)
C30	0.0180 (10)	0.0147 (10)	0.0248 (11)	0.0001 (8)	−0.0047 (8)	0.0022 (8)
C31	0.0162 (10)	0.0155 (10)	0.0249 (11)	0.0012 (8)	−0.0070 (8)	0.0008 (8)
C32	0.0206 (11)	0.0205 (11)	0.0298 (12)	−0.0032 (9)	−0.0090 (9)	−0.0011 (9)
C33	0.0251 (12)	0.0201 (11)	0.0266 (12)	−0.0006 (9)	−0.0097 (9)	−0.0057 (9)
C34	0.0221 (11)	0.0177 (10)	0.0213 (11)	0.0035 (8)	−0.0103 (8)	−0.0039 (8)
C35	0.0222 (11)	0.0209 (11)	0.0193 (10)	0.0043 (9)	−0.0085 (8)	−0.0065 (8)
C36	0.0184 (10)	0.0243 (11)	0.0164 (10)	0.0047 (8)	−0.0075 (8)	−0.0030 (8)
C37	0.0218 (11)	0.0323 (12)	0.0154 (10)	0.0048 (9)	−0.0037 (8)	−0.0048 (9)
C38	0.0215 (11)	0.0298 (12)	0.0178 (10)	0.0006 (9)	−0.0036 (8)	0.0005 (9)
C39	0.0157 (10)	0.0228 (11)	0.0164 (10)	0.0017 (8)	−0.0035 (8)	0.0014 (8)
C40	0.0147 (10)	0.0217 (11)	0.0206 (10)	−0.0004 (8)	−0.0019 (8)	0.0010 (8)
C25A	0.0273 (12)	0.0214 (11)	0.0176 (10)	−0.0017 (9)	−0.0101 (9)	−0.0004 (8)
C25B	0.0447 (15)	0.0232 (12)	0.0200 (11)	0.0010 (10)	−0.0091 (10)	−0.0032 (9)
C25C	0.0491 (16)	0.0284 (13)	0.0191 (11)	−0.0073 (11)	−0.0038 (11)	−0.0048 (10)
C25D	0.0497 (17)	0.0375 (15)	0.0196 (12)	−0.0100 (12)	0.0005 (11)	−0.0025 (10)
C30A	0.0197 (11)	0.0218 (11)	0.0267 (12)	−0.0042 (9)	−0.0021 (9)	−0.0006 (9)
C30B	0.0196 (11)	0.0245 (11)	0.0284 (12)	−0.0030 (9)	−0.0004 (9)	−0.0040 (9)
C30C	0.0226 (12)	0.0337 (14)	0.0375 (14)	−0.0016 (10)	0.0029 (10)	−0.0042 (11)
C30D	0.0265 (13)	0.0418 (15)	0.0398 (15)	0.0041 (11)	−0.0038 (11)	0.0073 (12)
C40A	0.0216 (11)	0.0226 (11)	0.0193 (10)	−0.0041 (9)	−0.0002 (8)	0.0024 (9)
C40B	0.0268 (12)	0.0243 (11)	0.0208 (11)	−0.0028 (9)	−0.0042 (9)	−0.0025 (9)
C40C	0.0260 (12)	0.0279 (12)	0.0271 (12)	0.0012 (10)	−0.0043 (9)	0.0000 (10)
C40D	0.0323 (13)	0.0241 (12)	0.0318 (13)	−0.0045 (10)	−0.0050 (10)	0.0040 (10)

### Geometric parameters (Å, º)

**Table:**

Ni1—N21	1.9089 (17)	Ni2—N25	1.9044 (17)
Ni1—N22	1.9107 (17)	Ni2—N26	1.9062 (17)
Ni1—N24	1.9170 (17)	Ni2—N27	1.9128 (17)
Ni1—N23	1.9176 (17)	Ni2—N28	1.9147 (17)
N21—C1	1.378 (3)	N25—C21	1.378 (3)
N21—C4	1.388 (3)	N25—C24	1.387 (3)
N22—C6	1.383 (3)	N26—C29	1.383 (3)
N22—C9	1.383 (3)	N26—C26	1.384 (3)
N23—C11	1.379 (3)	N27—C34	1.379 (3)
N23—C14	1.383 (3)	N27—C31	1.388 (3)
N24—C16	1.376 (3)	N28—C36	1.377 (3)
N24—C19	1.388 (3)	N28—C39	1.388 (3)
C1—C20	1.394 (3)	C21—C40	1.391 (3)
C1—C2	1.439 (3)	C21—C22	1.443 (3)
C2—C3	1.346 (3)	C22—C23	1.344 (3)
C2—H2A	0.9500	C22—H22A	0.9500
C3—C4	1.434 (3)	C23—C24	1.434 (3)
C3—H3A	0.9500	C23—H23A	0.9500
C4—C5	1.387 (3)	C24—C25	1.391 (3)
C5—C6	1.392 (3)	C25—C26	1.389 (3)
C5—C5A	1.516 (3)	C25—C25A	1.510 (3)
C6—C7	1.433 (3)	C26—C27	1.436 (3)
C7—C8	1.350 (3)	C27—C28	1.352 (3)
C7—H7A	0.9500	C27—H27A	0.9500
C8—C9	1.428 (3)	C28—C29	1.431 (3)
C8—H8A	0.9500	C28—H28A	0.9500
C9—C10	1.400 (3)	C29—C30	1.400 (3)
C10—C11	1.385 (3)	C30—C31	1.381 (3)
C10—C10A	1.515 (3)	C30—C30A	1.515 (3)
C11—C12	1.444 (3)	C31—C32	1.442 (3)
C12—C13	1.347 (3)	C32—C33	1.342 (3)
C12—H12A	0.9500	C32—H32A	0.9500
C13—C14	1.430 (3)	C33—C34	1.427 (3)
C13—H13A	0.9500	C33—H33A	0.9500
C14—C15	1.371 (3)	C34—C35	1.379 (3)
C15—C16	1.376 (3)	C35—C36	1.374 (3)
C15—H15A	0.9500	C35—H35A	0.9500
C16—C17	1.428 (3)	C36—C37	1.433 (3)
C17—C18	1.345 (3)	C37—C38	1.347 (3)
C17—H17A	0.9500	C37—H37A	0.9500
C18—C19	1.443 (3)	C38—C39	1.442 (3)
C18—H18A	0.9500	C38—H38A	0.9500
C19—C20	1.385 (3)	C39—C40	1.389 (3)
C20—C20A	1.516 (3)	C40—C40A	1.513 (3)
C5A—C5B	1.531 (3)	C25A—C25B	1.531 (3)
C5A—H5AA	0.9900	C25A—H25A	0.9900
C5A—H5AB	0.9900	C25A—H25B	0.9900
C5B—C5C	1.525 (3)	C25B—C25C	1.530 (3)
C5B—H5BA	0.9900	C25B—H25C	0.9900
C5B—H5BB	0.9900	C25B—H25D	0.9900
C5C—C5D	1.520 (4)	C25C—C25D	1.511 (3)
C5C—H5CA	0.9900	C25C—H25E	0.9900
C5C—H5CB	0.9900	C25C—H25F	0.9900
C5D—H5DA	0.9800	C25D—H25G	0.9800
C5D—H5DB	0.9800	C25D—H25H	0.9800
C5D—H5DC	0.9800	C25D—H25I	0.9800
C10A—C10B	1.531 (3)	C30A—C30B	1.535 (3)
C10A—H10A	0.9900	C30A—H30A	0.9900
C10A—H10B	0.9900	C30A—H30B	0.9900
C10B—C10D	1.522 (3)	C30B—C30D	1.516 (3)
C10B—C10C	1.526 (3)	C30B—C30C	1.526 (3)
C10B—H10C	1.0000	C30B—H30C	1.0000
C10C—H10D	0.9800	C30C—H30D	0.9800
C10C—H10E	0.9800	C30C—H30E	0.9800
C10C—H10F	0.9800	C30C—H30F	0.9800
C10D—H10G	0.9800	C30D—H30G	0.9800
C10D—H10H	0.9800	C30D—H30H	0.9800
C10D—H10I	0.9800	C30D—H30I	0.9800
C20A—C20B	1.538 (3)	C40A—C40B	1.535 (3)
C20A—H20A	0.9900	C40A—H40A	0.9900
C20A—H20B	0.9900	C40A—H40B	0.9900
C20B—C20D	1.525 (3)	C40B—C40C	1.525 (3)
C20B—C20C	1.522 (3)	C40B—C40D	1.537 (3)
C20B—H20C	1.0000	C40B—H40C	1.0000
C20C—H20D	0.9800	C40C—H40D	0.9800
C20C—H20E	0.9800	C40C—H40E	0.9800
C20C—H20F	0.9800	C40C—H40F	0.9800
C20D—H20G	0.9800	C40D—H40G	0.9800
C20D—H20H	0.9800	C40D—H40H	0.9800
C20D—H20I	0.9800	C40D—H40I	0.9800
			
N21—Ni1—N22	90.66 (7)	N25—Ni2—N26	90.61 (7)
N21—Ni1—N24	89.63 (7)	N25—Ni2—N27	179.48 (7)
N22—Ni1—N24	178.74 (7)	N26—Ni2—N27	89.38 (7)
N21—Ni1—N23	179.09 (7)	N25—Ni2—N28	89.50 (7)
N22—Ni1—N23	89.17 (7)	N26—Ni2—N28	179.69 (7)
N24—Ni1—N23	90.56 (7)	N27—Ni2—N28	90.51 (7)
C1—N21—C4	105.34 (16)	C21—N25—C24	105.49 (17)
C1—N21—Ni1	127.44 (14)	C21—N25—Ni2	127.18 (14)
C4—N21—Ni1	127.21 (14)	C24—N25—Ni2	127.32 (14)
C6—N22—C9	105.75 (17)	C29—N26—C26	105.99 (17)
C6—N22—Ni1	126.81 (14)	C29—N26—Ni2	127.06 (14)
C9—N22—Ni1	127.41 (14)	C26—N26—Ni2	126.95 (14)
C11—N23—C14	105.22 (17)	C34—N27—C31	105.14 (17)
C11—N23—Ni1	128.42 (14)	C34—N27—Ni2	126.75 (14)
C14—N23—Ni1	126.36 (15)	C31—N27—Ni2	128.11 (14)
C16—N24—C19	105.63 (17)	C36—N28—C39	105.69 (17)
C16—N24—Ni1	126.66 (15)	C36—N28—Ni2	126.53 (14)
C19—N24—Ni1	127.67 (14)	C39—N28—Ni2	127.79 (14)
N21—C1—C20	126.08 (19)	N25—C21—C40	126.20 (19)
N21—C1—C2	110.00 (18)	N25—C21—C22	109.76 (18)
C20—C1—C2	123.73 (19)	C40—C21—C22	123.74 (19)
C3—C2—C1	107.32 (19)	C23—C22—C21	107.39 (19)
C3—C2—H2A	126.3	C23—C22—H22A	126.3
C1—C2—H2A	126.3	C21—C22—H22A	126.3
C2—C3—C4	107.20 (19)	C22—C23—C24	107.22 (19)
C2—C3—H3A	126.4	C22—C23—H23A	126.4
C4—C3—H3A	126.4	C24—C23—H23A	126.4
C5—C4—N21	124.80 (19)	N25—C24—C25	124.76 (19)
C5—C4—C3	124.75 (19)	N25—C24—C23	110.01 (18)
N21—C4—C3	110.02 (18)	C25—C24—C23	124.82 (19)
C4—C5—C6	121.28 (19)	C26—C25—C24	121.27 (19)
C4—C5—C5A	119.47 (19)	C26—C25—C25A	118.97 (19)
C6—C5—C5A	118.81 (19)	C24—C25—C25A	119.13 (19)
N22—C6—C5	125.59 (19)	N26—C26—C25	125.34 (19)
N22—C6—C7	109.69 (18)	N26—C26—C27	109.56 (18)
C5—C6—C7	124.32 (19)	C25—C26—C27	124.62 (19)
C8—C7—C6	107.20 (19)	C28—C27—C26	107.15 (19)
C8—C7—H7A	126.4	C28—C27—H27A	126.4
C6—C7—H7A	126.4	C26—C27—H27A	126.4
C7—C8—C9	107.54 (19)	C27—C28—C29	107.59 (19)
C7—C8—H8A	126.2	C27—C28—H28A	126.2
C9—C8—H8A	126.2	C29—C28—H28A	126.2
N22—C9—C10	125.45 (19)	N26—C29—C30	125.74 (19)
N22—C9—C8	109.73 (18)	N26—C29—C28	109.59 (18)
C10—C9—C8	124.4 (2)	C30—C29—C28	124.3 (2)
C11—C10—C9	120.3 (2)	C31—C30—C29	120.24 (19)
C11—C10—C10A	120.88 (19)	C31—C30—C30A	121.19 (19)
C9—C10—C10A	118.7 (2)	C29—C30—C30A	118.46 (19)
N23—C11—C10	124.79 (19)	C30—C31—N27	124.45 (19)
N23—C11—C12	109.91 (19)	C30—C31—C32	125.4 (2)
C10—C11—C12	125.0 (2)	N27—C31—C32	109.63 (19)
C13—C12—C11	107.1 (2)	C33—C32—C31	107.2 (2)
C13—C12—H12A	126.4	C33—C32—H32A	126.4
C11—C12—H12A	126.4	C31—C32—H32A	126.4
C12—C13—C14	107.1 (2)	C32—C33—C34	107.4 (2)
C12—C13—H13A	126.5	C32—C33—H33A	126.3
C14—C13—H13A	126.5	C34—C33—H33A	126.3
C15—C14—N23	124.62 (19)	C35—C34—N27	124.3 (2)
C15—C14—C13	124.2 (2)	C35—C34—C33	124.6 (2)
N23—C14—C13	110.51 (19)	N27—C34—C33	110.51 (19)
C14—C15—C16	122.8 (2)	C36—C35—C34	123.0 (2)
C14—C15—H15A	118.6	C36—C35—H35A	118.5
C16—C15—H15A	118.6	C34—C35—H35A	118.5
C15—C16—N24	124.9 (2)	C35—C36—N28	124.67 (19)
C15—C16—C17	124.3 (2)	C35—C36—C37	124.7 (2)
N24—C16—C17	110.40 (19)	N28—C36—C37	110.19 (19)
C18—C17—C16	107.3 (2)	C38—C37—C36	107.3 (2)
C18—C17—H17A	126.4	C38—C37—H37A	126.3
C16—C17—H17A	126.4	C36—C37—H37A	126.3
C17—C18—C19	107.4 (2)	C37—C38—C39	107.28 (19)
C17—C18—H18A	126.3	C37—C38—H38A	126.4
C19—C18—H18A	126.3	C39—C38—H38A	126.4
C20—C19—N24	124.78 (19)	N28—C39—C40	124.70 (19)
C20—C19—C18	125.5 (2)	N28—C39—C38	109.42 (18)
N24—C19—C18	109.19 (19)	C40—C39—C38	125.3 (2)
C19—C20—C1	120.25 (19)	C21—C40—C39	119.68 (19)
C19—C20—C20A	120.60 (19)	C21—C40—C40A	119.78 (19)
C1—C20—C20A	119.02 (19)	C39—C40—C40A	120.45 (19)
C5—C5A—C5B	113.49 (18)	C25—C25A—C25B	112.54 (18)
C5—C5A—H5AA	108.9	C25—C25A—H25A	109.1
C5B—C5A—H5AA	108.9	C25B—C25A—H25A	109.1
C5—C5A—H5AB	108.9	C25—C25A—H25B	109.1
C5B—C5A—H5AB	108.9	C25B—C25A—H25B	109.1
H5AA—C5A—H5AB	107.7	H25A—C25A—H25B	107.8
C5C—C5B—C5A	112.96 (19)	C25C—C25B—C25A	113.5 (2)
C5C—C5B—H5BA	109.0	C25C—C25B—H25C	108.9
C5A—C5B—H5BA	109.0	C25A—C25B—H25C	108.9
C5C—C5B—H5BB	109.0	C25C—C25B—H25D	108.9
C5A—C5B—H5BB	109.0	C25A—C25B—H25D	108.9
H5BA—C5B—H5BB	107.8	H25C—C25B—H25D	107.7
C5D—C5C—C5B	112.5 (2)	C25D—C25C—C25B	113.8 (2)
C5D—C5C—H5CA	109.1	C25D—C25C—H25E	108.8
C5B—C5C—H5CA	109.1	C25B—C25C—H25E	108.8
C5D—C5C—H5CB	109.1	C25D—C25C—H25F	108.8
C5B—C5C—H5CB	109.1	C25B—C25C—H25F	108.8
H5CA—C5C—H5CB	107.8	H25E—C25C—H25F	107.7
C5C—C5D—H5DA	109.5	C25C—C25D—H25G	109.5
C5C—C5D—H5DB	109.5	C25C—C25D—H25H	109.5
H5DA—C5D—H5DB	109.5	H25G—C25D—H25H	109.5
C5C—C5D—H5DC	109.5	C25C—C25D—H25I	109.5
H5DA—C5D—H5DC	109.5	H25G—C25D—H25I	109.5
H5DB—C5D—H5DC	109.5	H25H—C25D—H25I	109.5
C10—C10A—C10B	113.14 (18)	C30—C30A—C30B	112.90 (18)
C10—C10A—H10A	109.0	C30—C30A—H30A	109.0
C10B—C10A—H10A	109.0	C30B—C30A—H30A	109.0
C10—C10A—H10B	109.0	C30—C30A—H30B	109.0
C10B—C10A—H10B	109.0	C30B—C30A—H30B	109.0
H10A—C10A—H10B	107.8	H30A—C30A—H30B	107.8
C10D—C10B—C10C	110.9 (2)	C30D—C30B—C30C	111.5 (2)
C10D—C10B—C10A	109.87 (19)	C30D—C30B—C30A	112.1 (2)
C10C—C10B—C10A	111.9 (2)	C30C—C30B—C30A	109.69 (19)
C10D—C10B—H10C	108.0	C30D—C30B—H30C	107.8
C10C—C10B—H10C	108.0	C30C—C30B—H30C	107.8
C10A—C10B—H10C	108.0	C30A—C30B—H30C	107.8
C10B—C10C—H10D	109.5	C30B—C30C—H30D	109.5
C10B—C10C—H10E	109.5	C30B—C30C—H30E	109.5
H10D—C10C—H10E	109.5	H30D—C30C—H30E	109.5
C10B—C10C—H10F	109.5	C30B—C30C—H30F	109.5
H10D—C10C—H10F	109.5	H30D—C30C—H30F	109.5
H10E—C10C—H10F	109.5	H30E—C30C—H30F	109.5
C10B—C10D—H10G	109.5	C30B—C30D—H30G	109.5
C10B—C10D—H10H	109.5	C30B—C30D—H30H	109.5
H10G—C10D—H10H	109.5	H30G—C30D—H30H	109.5
C10B—C10D—H10I	109.5	C30B—C30D—H30I	109.5
H10G—C10D—H10I	109.5	H30G—C30D—H30I	109.5
H10H—C10D—H10I	109.5	H30H—C30D—H30I	109.5
C20—C20A—C20B	114.66 (18)	C40—C40A—C40B	114.43 (18)
C20—C20A—H20A	108.6	C40—C40A—H40A	108.7
C20B—C20A—H20A	108.6	C40B—C40A—H40A	108.7
C20—C20A—H20B	108.6	C40—C40A—H40B	108.7
C20B—C20A—H20B	108.6	C40B—C40A—H40B	108.7
H20A—C20A—H20B	107.6	H40A—C40A—H40B	107.6
C20D—C20B—C20C	110.9 (2)	C40C—C40B—C40A	113.18 (19)
C20D—C20B—C20A	108.64 (19)	C40C—C40B—C40D	110.34 (19)
C20C—C20B—C20A	112.7 (2)	C40A—C40B—C40D	108.76 (18)
C20D—C20B—H20C	108.2	C40C—C40B—H40C	108.1
C20C—C20B—H20C	108.2	C40A—C40B—H40C	108.1
C20A—C20B—H20C	108.2	C40D—C40B—H40C	108.1
C20B—C20C—H20D	109.5	C40B—C40C—H40D	109.5
C20B—C20C—H20E	109.5	C40B—C40C—H40E	109.5
H20D—C20C—H20E	109.5	H40D—C40C—H40E	109.5
C20B—C20C—H20F	109.5	C40B—C40C—H40F	109.5
H20D—C20C—H20F	109.5	H40D—C40C—H40F	109.5
H20E—C20C—H20F	109.5	H40E—C40C—H40F	109.5
C20B—C20D—H20G	109.5	C40B—C40D—H40G	109.5
C20B—C20D—H20H	109.5	C40B—C40D—H40H	109.5
H20G—C20D—H20H	109.5	H40G—C40D—H40H	109.5
C20B—C20D—H20I	109.5	C40B—C40D—H40I	109.5
H20G—C20D—H20I	109.5	H40G—C40D—H40I	109.5
H20H—C20D—H20I	109.5	H40H—C40D—H40I	109.5
			
N22—Ni1—N21—C1	−162.73 (17)	N26—Ni2—N25—C21	161.29 (17)
N24—Ni1—N21—C1	16.04 (17)	N28—Ni2—N25—C21	−19.00 (17)
N22—Ni1—N21—C4	18.99 (17)	N26—Ni2—N25—C24	−18.48 (17)
N24—Ni1—N21—C4	−162.24 (17)	N28—Ni2—N25—C24	161.24 (17)
N21—Ni1—N22—C6	−16.75 (17)	N25—Ni2—N26—C29	−160.60 (17)
N23—Ni1—N22—C6	162.36 (17)	N27—Ni2—N26—C29	19.93 (17)
N21—Ni1—N22—C9	161.03 (17)	N25—Ni2—N26—C26	18.11 (17)
N23—Ni1—N22—C9	−19.86 (17)	N27—Ni2—N26—C26	−161.36 (17)
N22—Ni1—N23—C11	18.90 (18)	N26—Ni2—N27—C34	160.36 (17)
N24—Ni1—N23—C11	−159.87 (18)	N28—Ni2—N27—C34	−19.36 (17)
N22—Ni1—N23—C14	−161.52 (17)	N26—Ni2—N27—C31	−21.01 (17)
N24—Ni1—N23—C14	19.71 (17)	N28—Ni2—N27—C31	159.28 (17)
N21—Ni1—N24—C16	162.10 (17)	N25—Ni2—N28—C36	−159.91 (17)
N23—Ni1—N24—C16	−17.01 (17)	N27—Ni2—N28—C36	19.57 (17)
N21—Ni1—N24—C19	−20.39 (17)	N25—Ni2—N28—C39	19.96 (17)
N23—Ni1—N24—C19	160.49 (17)	N27—Ni2—N28—C39	−160.56 (17)
C4—N21—C1—C20	175.2 (2)	C24—N25—C21—C40	−173.9 (2)
Ni1—N21—C1—C20	−3.3 (3)	Ni2—N25—C21—C40	6.3 (3)
C4—N21—C1—C2	0.1 (2)	C24—N25—C21—C22	0.0 (2)
Ni1—N21—C1—C2	−178.46 (14)	Ni2—N25—C21—C22	−179.78 (14)
N21—C1—C2—C3	2.0 (3)	N25—C21—C22—C23	−2.3 (3)
C20—C1—C2—C3	−173.2 (2)	C40—C21—C22—C23	171.7 (2)
C1—C2—C3—C4	−3.2 (2)	C21—C22—C23—C24	3.6 (3)
C1—N21—C4—C5	170.7 (2)	C21—N25—C24—C25	−170.7 (2)
Ni1—N21—C4—C5	−10.8 (3)	Ni2—N25—C24—C25	9.1 (3)
C1—N21—C4—C3	−2.1 (2)	C21—N25—C24—C23	2.2 (2)
Ni1—N21—C4—C3	176.46 (14)	Ni2—N25—C24—C23	−178.00 (14)
C2—C3—C4—C5	−169.3 (2)	C22—C23—C24—N25	−3.7 (3)
C2—C3—C4—N21	3.5 (2)	C22—C23—C24—C25	169.2 (2)
N21—C4—C5—C6	−7.1 (3)	N25—C24—C25—C26	8.1 (3)
C3—C4—C5—C6	164.7 (2)	C23—C24—C25—C26	−163.8 (2)
N21—C4—C5—C5A	−179.42 (19)	N25—C24—C25—C25A	178.94 (19)
C3—C4—C5—C5A	−7.7 (3)	C23—C24—C25—C25A	7.0 (3)
C9—N22—C6—C5	−172.0 (2)	C29—N26—C26—C25	170.5 (2)
Ni1—N22—C6—C5	6.2 (3)	Ni2—N26—C26—C25	−8.4 (3)
C9—N22—C6—C7	1.0 (2)	C29—N26—C26—C27	−1.8 (2)
Ni1—N22—C6—C7	179.15 (14)	Ni2—N26—C26—C27	179.26 (14)
C4—C5—C6—N22	9.4 (3)	C24—C25—C26—N26	−8.5 (3)
C5A—C5—C6—N22	−178.18 (19)	C25A—C25—C26—N26	−179.28 (19)
C4—C5—C6—C7	−162.6 (2)	C24—C25—C26—C27	162.7 (2)
C5A—C5—C6—C7	9.8 (3)	C25A—C25—C26—C27	−8.1 (3)
N22—C6—C7—C8	−2.6 (2)	N26—C26—C27—C28	3.4 (2)
C5—C6—C7—C8	170.5 (2)	C25—C26—C27—C28	−169.0 (2)
C6—C7—C8—C9	3.1 (2)	C26—C27—C28—C29	−3.5 (2)
C6—N22—C9—C10	−172.2 (2)	C26—N26—C29—C30	173.1 (2)
Ni1—N22—C9—C10	9.7 (3)	Ni2—N26—C29—C30	−7.9 (3)
C6—N22—C9—C8	0.9 (2)	C26—N26—C29—C28	−0.4 (2)
Ni1—N22—C9—C8	−177.23 (14)	Ni2—N26—C29—C28	178.55 (14)
C7—C8—C9—N22	−2.6 (2)	C27—C28—C29—N26	2.5 (2)
C7—C8—C9—C10	170.6 (2)	C27—C28—C29—C30	−171.1 (2)
N22—C9—C10—C11	10.1 (3)	N26—C29—C30—C31	−11.9 (3)
C8—C9—C10—C11	−162.0 (2)	C28—C29—C30—C31	160.7 (2)
N22—C9—C10—C10A	−173.86 (19)	N26—C29—C30—C30A	171.94 (19)
C8—C9—C10—C10A	14.0 (3)	C28—C29—C30—C30A	−15.5 (3)
C14—N23—C11—C10	173.2 (2)	C29—C30—C31—N27	10.9 (3)
Ni1—N23—C11—C10	−7.1 (3)	C30A—C30—C31—N27	−172.96 (19)
C14—N23—C11—C12	−0.5 (2)	C29—C30—C31—C32	−160.1 (2)
Ni1—N23—C11—C12	179.20 (14)	C30A—C30—C31—C32	16.0 (3)
C9—C10—C11—N23	−11.3 (3)	C34—N27—C31—C30	−171.49 (19)
C10A—C10—C11—N23	172.68 (19)	Ni2—N27—C31—C30	9.6 (3)
C9—C10—C11—C12	161.4 (2)	C34—N27—C31—C32	0.7 (2)
C10A—C10—C11—C12	−14.6 (3)	Ni2—N27—C31—C32	−178.14 (14)
N23—C11—C12—C13	2.7 (3)	C30—C31—C32—C33	169.5 (2)
C10—C11—C12—C13	−170.9 (2)	N27—C31—C32—C33	−2.7 (2)
C11—C12—C13—C14	−3.8 (3)	C31—C32—C33—C34	3.4 (2)
C11—N23—C14—C15	168.8 (2)	C31—N27—C34—C35	−169.9 (2)
Ni1—N23—C14—C15	−10.9 (3)	Ni2—N27—C34—C35	9.0 (3)
C11—N23—C14—C13	−1.9 (2)	C31—N27—C34—C33	1.4 (2)
Ni1—N23—C14—C13	178.43 (14)	Ni2—N27—C34—C33	−179.73 (14)
C12—C13—C14—C15	−167.0 (2)	C32—C33—C34—C35	168.1 (2)
C12—C13—C14—N23	3.7 (3)	C32—C33—C34—N27	−3.1 (2)
N23—C14—C15—C16	−8.7 (3)	N27—C34—C35—C36	9.3 (3)
C13—C14—C15—C16	160.8 (2)	C33—C34—C35—C36	−160.7 (2)
C14—C15—C16—N24	11.7 (3)	C34—C35—C36—N28	−9.1 (3)
C14—C15—C16—C17	−160.6 (2)	C34—C35—C36—C37	162.5 (2)
C19—N24—C16—C15	−172.9 (2)	C39—N28—C36—C35	170.6 (2)
Ni1—N24—C16—C15	5.1 (3)	Ni2—N28—C36—C35	−9.5 (3)
C19—N24—C16—C17	0.4 (2)	C39—N28—C36—C37	−2.0 (2)
Ni1—N24—C16—C17	178.33 (14)	Ni2—N28—C36—C37	177.93 (14)
C15—C16—C17—C18	170.5 (2)	C35—C36—C37—C38	−169.2 (2)
N24—C16—C17—C18	−2.8 (3)	N28—C36—C37—C38	3.4 (2)
C16—C17—C18—C19	3.9 (3)	C36—C37—C38—C39	−3.2 (2)
C16—N24—C19—C20	−169.7 (2)	C36—N28—C39—C40	171.8 (2)
Ni1—N24—C19—C20	12.4 (3)	Ni2—N28—C39—C40	−8.1 (3)
C16—N24—C19—C18	2.0 (2)	C36—N28—C39—C38	0.0 (2)
Ni1—N24—C19—C18	−175.91 (14)	Ni2—N28—C39—C38	−179.94 (14)
C17—C18—C19—C20	167.8 (2)	C37—C38—C39—N28	2.1 (2)
C17—C18—C19—N24	−3.8 (2)	C37—C38—C39—C40	−169.6 (2)
N24—C19—C20—C1	7.7 (3)	N25—C21—C40—C39	13.6 (3)
C18—C19—C20—C1	−162.7 (2)	C22—C21—C40—C39	−159.5 (2)
N24—C19—C20—C20A	−176.53 (19)	N25—C21—C40—C40A	−169.83 (19)
C18—C19—C20—C20A	13.1 (3)	C22—C21—C40—C40A	17.1 (3)
N21—C1—C20—C19	−12.4 (3)	N28—C39—C40—C21	−12.5 (3)
C2—C1—C20—C19	162.1 (2)	C38—C39—C40—C21	158.0 (2)
N21—C1—C20—C20A	171.77 (19)	N28—C39—C40—C40A	170.88 (19)
C2—C1—C20—C20A	−13.7 (3)	C38—C39—C40—C40A	−18.6 (3)
C4—C5—C5A—C5B	88.9 (2)	C26—C25—C25A—C25B	85.4 (2)
C6—C5—C5A—C5B	−83.6 (2)	C24—C25—C25A—C25B	−85.6 (2)
C5—C5A—C5B—C5C	175.75 (19)	C25—C25A—C25B—C25C	−178.5 (2)
C5A—C5B—C5C—C5D	−175.8 (2)	C25A—C25B—C25C—C25D	65.3 (3)
C11—C10—C10A—C10B	−106.1 (2)	C31—C30—C30A—C30B	112.5 (2)
C9—C10—C10A—C10B	77.8 (3)	C29—C30—C30A—C30B	−71.3 (3)
C10—C10A—C10B—C10D	−173.6 (2)	C30—C30A—C30B—C30D	−63.3 (3)
C10—C10A—C10B—C10C	62.8 (3)	C30—C30A—C30B—C30C	172.2 (2)
C19—C20—C20A—C20B	112.8 (2)	C21—C40—C40A—C40B	63.0 (3)
C1—C20—C20A—C20B	−71.3 (3)	C39—C40—C40A—C40B	−120.4 (2)
C20—C20A—C20B—C20D	−173.34 (19)	C40—C40A—C40B—C40C	44.7 (3)
C20—C20A—C20B—C20C	−50.1 (3)	C40—C40A—C40B—C40D	167.76 (19)
